# Child marriage and associated outcomes in northern Ghana: a cross-sectional study

**DOI:** 10.1186/s12889-018-5166-6

**Published:** 2018-02-26

**Authors:** Richard de Groot, Maxwell Yiryele Kuunyem, Tia Palermo, on behalf of the Ghana LEAP 1000 evaluation team, Richard de Groot, Tia Palermo, Isaac Osei-Akoto, Clement Adamba, Joseph K. Darko, Robert Darko Osei, Francis Dompae, Nana Yaw, Clare Barrington, Sara Abdoulayi, Gustavo Angeles, Averi Chakrabarti, Frank Otchere, Akalpa J. Akaligaung, Raymond Aborigo, Rachel Kidman, Annalisa Caparello

**Affiliations:** 1 UNICEF Office of Research—Innocenti, Piazza SS. Annunziata 12, 50122 Florence, Italy; 2 UNICEF Ghana, 4-8th Rangoon Close, P.O. Box 5051, Accra-North, Ghana

**Keywords:** Child marriage, women’s health, Ghana, Sub-Saharan Africa

## Abstract

**Background:**

Child marriage is a human rights violation disproportionately affecting girls in lower- and middle-income countries and has serious public health implications. In Ghana, one in five girls marry before their 18th birthday and one in 20 girls is married before her 15th birthday. This paper uses a unique dataset from Northern Ghana to examine the association between child marriage and adverse outcomes for women among a uniquely vulnerable population.

**Methods:**

Baseline data from on ongoing impact evaluation of a government-run cash transfer programme was used. The sample consisted of 1349 ever-married women aged 20–29 years from 2497 households in the Northern and Upper East regions of Ghana. We estimated a series of ordinary least squares (OLS) and logistic regression models to examine associations of child marriage with health, fertility, contraception, child mortality, social support, stress and agency outcomes among women, controlling for individual characteristics and household-level factors.

**Results:**

Child marriage in this sample was associated with increased odds of poorer health, as measured by difficulties in daily activities (OR = 2.08; CI 1.28–3.38 among women 20–24 years and OR = 1.58; CI 1.19–2.12 among women 20–29 years), increased odds of child mortality among first-born children (OR = 2.03; CI 1.09–3.77 among women 20–24 years) and lower odds of believing that one’s life is determined by their own actions (OR = 0.42; CI 0.25–0.72 among women 20–24 years and OR = 0.54; CI 0.39–0.75 among women 20–29 years). Conversely, child marriage was associated with lower levels of reported stress (regression coefficient = − 1.18; CI -1.84–-0.51 among women 20–29 years).

**Conclusions:**

Child marriage is common in Northern Ghana and is associated with poor health, increased child mortality, and low agency among women in this sample of extremely poor households. While not much is known about effective measures to combat child marriage in the context of Ghana, programmes that address key drivers of early marriage such as economic insecurity and school enrolment at the secondary level, should be examined with respect to their effectiveness at reducing early marriage.

**Trial registration**: Registered in the Registry for International Development Impact Evaluations (RIDIE) on 01 July 2015, with number RIDIE-STUDY-ID-55942496d53af.

## Background

Child marriage is a human rights violation and has adverse effects on the children (overwhelmingly girls) who enter into these marriages, and on their future children, creating an intergenerational cycle of disadvantage. Sometimes referred to as early marriage, child marriage is defined as a marriage occurring when one of the spouses is younger than 18 years at the time of marriage or official union [1].

Rates of child marriage (typically measured as the share of women 20 to 24 years old who first married or entered into union before age 18) are highest in low-income countries [2]. Data from 2012 from 41 low-income countries indicated that the global rate of child marriage was 30%, with a high of 75% in Niger [2]. Even within low-income countries, disparities exist and child marriage is more common among girls in rural areas, those with low educational attainment and those in the poorest socioeconomic strata [3, 4]. Trends in sub-Saharan Africa over the past 20 years are not always encouraging; while the prevalence of child marriage has decreased in most countries, these decreases have stalled in many countries, including Niger, Malawi, Kenya and Senegal [5].

Factors that contribute to child marriage include economic drivers, such as incentives to marry out girls to reduce the financial burden on the household or receive the bride price [6]. Other factors include social norms and the need to reinforce social ties and perceived protection. That is, parents often believe that they may be able to improve their social status through their daughter’s marriage, linking two families together. Alternatively, parents may hold the belief that by marrying their daughter at an early age, she will be protected from sexual abuse, unwanted pregnancies and the risk of sexually transmitted infections [6, 7].

Negative health consequences of child marriage include poorer maternal and reproductive health, increased risk of HIV and other sexually transmitted infections, intimate partner violence and maternal mortality [7–10]. Moreover, early childbearing increases risks to women’s health, as maternal disorders^1^ including complications during pregnancy and child birth are the leading cause of death among women aged 20–24 years globally and the second leading cause among adolescent girls aged 15 to 19 years [11]. In addition, girls who marry early are at a higher risk of intimate partner violence, poorer mental health and limited decision making power due to power imbalances within their marriage [6, 9, 10]. Child marriage is also associated with adverse economic outcomes, including lower educational attainment, lower socioeconomic status, and higher rates of poverty [12]. Further, the adverse consequences of early marriage are intergenerational. Children born to women affected by child marriage have higher mortality rates, are more likely to be born prematurely or with low birthweight and have poorer health and nutritional status [13, 14].

### Ghanaian context

In Ghana, a lower-middle-income country, child marriage is prohibited under the constitution and by law. According to the Ghanaian constitution of 1992, any person under the age of 18 is a child and can therefore not marry or be married off. This is underscored by the 1998 Children’s Act, which sets the legal age of marriage at 18 for both boys and girls [15].

Despite this legal framework, the prevalence of child marriage in Ghana remains high, and one in five girls marries before her 18th birthday (20.7%) and one in 20 girls (4.9%) marries before her 15th birthday [16]. Although child marriage can occur among males as well, the prevalence is much lower: only 2.3% of men aged 20 to 24 years are married by age 18 in Ghana [16]. The highest prevalence of child marriage in Ghana occurs in the three Northern regions, where more than one in three girls marry before age 18. In addition, the child marriage rate is higher in rural areas, among the poorest population and among uneducated women. The aggregate rate of marriage before age 18 has been decreasing from 35% in the 1990s to 28% in 2003 and 25% in 2008, but remained stable between 2011 and 2014. However, data from the latest two nationally representative surveys shows that the rate of child marriage increased in the three Northern regions (from 26.4% in 2011 to 33.6% in 2014) and decreased in the other regions (from 20.9 to 18.5% and from 19.2 to 18.5% in the Central and Southern regions respectively), increasing disparities between the North and the rest of the country.^2^

Despite a large global evidence base on the consequences of child marriage, in-depth studies studying dynamics and consequences of child marriage in Ghana specifically are scarce. A better understanding of the dynamics around child marriage is important to motivate more action around reducing child marriage rates, particularly given that decreases have stalled in recent years, and to inform programming assisting child brides. In addition, existing datasets commonly used to study adverse impacts of child marriage, including Demographic and Health Surveys (DHS) and Multiple Indicator Cluster Surveys (MICS), aim to be comparable across countries and thus may not include all of the indicators of interest. Using a unique dataset from extremely poor households in two regions in Northern Ghana, the region identified as having the highest child marriage rates, this paper explores the relationship between child marriage and adverse outcomes among women and their children and also contributes to the literature by implementing innovative measures related to women’s empowerment, a concept of widespread interest in development but one that is notoriously hard to measure in all its dimensions.

More specifically, we examined the following research question: To what extent is child marriage associated with adverse outcomes, including general health, reproductive health, agency, social support, and stress? We first examined associations between child marriage and outcomes examined in the existing evidence base (including general health, reproductive health and child mortality) using our data to investigate whether these relationships also hold in this setting. Next, we improve upon the existing evidence base by testing the association of child marriage with other outcomes of interest. One such outcome is women’s empowerment, which Kabeer (1999) argued entails a process of change and defined the term as “the expansion in people’s ability to make strategic life choices in a context where this ability was previously denied to them” (p. 437). In this process, resources, agency and achievements are inter-related dimensions relating to the ability to exercise choice. In the current study, we focus on the agency dimension, using an innovative measure of agency based on the locus of control (described in more detail in the methods section). This is an improvement on commonly used measures of women’s empowerment, namely decision-making indices assessing women’s self-reported participation in typical household decisions. Such indices have limitations in their ability to capture empowerment and dynamics around decision-making [17].

Additionally, we assessed another innovative aspect of women’s empowerment: financial control and savings, which may reflect a woman’s higher bargaining power within the household [18], an outcome not commonly examined in existing studies on child marriage.

Finally, we examined measures related to social support and stress. Women who marry early may have lower levels of social support for a variety of reasons, for example by being forced to move in with the husband’s family, away from their peers in school, limiting access to social networks and friends [7, 19]. In addition, stress is both an important indicator of well-being in its own right [20, 21] but also a hypothesized pathway through which poverty and adverse life events may contribute to adverse outcomes [22]. In the context studied here, early marriage may induce stress as females newly navigate partnerships, new duties, childbearing and possibly violence. Further, given that poverty is a driver of child marriage [2, 6, 7, 19, 23], and poverty is strongly associated with stress and poor mental health, [24–26], early marriage, poverty and stress may be mutually reinforcing.

Informed by the evidence to date, we hypothesized that child marriage is associated with lower levels of general health and worse reproductive health outcomes, including higher likelihood of adolescence childbearing and increased child mortality.^3^ We further hypothesized that child marriage is associated with lower levels of agency, lower levels of social support and higher levels of self-perceived stress.

## Methods

### Sample

Data for this study come from the baseline survey of a longitudinal impact evaluation of the Livelihood for Empowerment Against Poverty (LEAP) 1000 programme. This is a government-run unconditional cash transfer programme targeted to pregnant women and women with children under the age of 15 months in extremely poor households, aimed at reducing poverty, increasing consumption and improving child nutrition. The programme was first rolled out in ten districts in Northern Ghana (three districts in Upper East region and seven in Northern region), and communities within these districts were targeted based on official poverty rankings established at district level. UNICEF Office of Research – Innocenti in collaboration with the Institute of Statistical, Social and Economic Research (ISSER) at the University of Ghana and the University of North Carolina at Chapel Hill (USA) carried out an impact evaluation of the LEAP 1000 roll-out in five of the 10 implementing districts between 2015 and 2017. Study eligibility criteria reflects the programme targeting, namely pregnant women and women (ages 15 to 49) with children under the age of 15 months in poor, rural households living in the Northern or Upper East region of Ghana. Households surveys were administered to the LEAP 1000 eligible woman (referred to as the “main respondent”) in each selected household.

Data used in the current study come from the baseline survey of this impact evaluation, which was administered from July through September 2015 to 2497 women who met the eligibility criteria.^4^ Respondents and their households were quite homogenous in terms of socio-economic status, with approximately 90% of the sample living below the national poverty line of US$ 1.83 per adult per day [27].^5^ The typical age group for studying child marriage is among ever-married women 20 to 24 years old [2, 28]. However, since this age range does not provide a sufficient sample for analysis in our data, we also use the broader age range of ever-married 20 to 29 years old for analyses in the current sample. These restrictions result in an analysis sample of 594 women 20 to 24 years old and 1349 total women 20 to 29 years old (Table 1).

**Table 1:** Sample characteristics, Women aged 20–29 years, Ghana LEAP 1000 Baseline

	Ever-married women 20 to 24 years old			Ever-married women 20 to 29 years old	
	N	%		N	%
Age					
20 to 24 years	594	100.0		594	44.0
25 to 29 years				755	56.0
Educational achievement					
No schooling	407	68.5		1000	74.1
Some primary	50	8.4		117	8.7
Primary completed or higher	137	23.1		232	17.2
Region of residence					
Northern	414	69.7		976	72.4
Upper East	180	30.3		373	27.7
Marital status					
Married/Union – Monogamous	441	74.2		913	67.7
Married/Union – Polygamous	148	24.9		418	31.0
Separated/Divorced/Widowed	5	0.8		18	1.3
Child marriage status					
Married after 18th birthday	416	70.0		1018	75.5
Married between 15 – 18th birthday	154	25.9		284	21.1
Married before 15th birthday	24	4.0		47	3.5

### Measures

The main independent variable in this analysis, child marriage, is based on the self-reported age at which the respondent got married or started living together with a partner for the first time. Women reporting marriage or cohabitation before the age of 18 were defined as experiencing child marriage.

Health outcomes examined included recent illness (defined as any illness in the last 14 days) and whether the woman reports having a valid National Health Insurance Scheme (NHIS) card (yes/no).^6^ Subjective health was assessed through a self-reported question with five response options (poor, fair, good, very good and excellent), which has been found to be a good predictor of future mortality and morbidity [29]. We constructed a dummy indicator equal to one if a respondent reported poor or fair health and equal to zero otherwise. A second subjective health outcome was created from a question asking whether the respondent believed her health was better than one year ago (yes/no). The final health outcome assessed difficulties with activities of daily living (ADL; items include carrying a 10 kg load for 500 m; bend, squat or kneel; and walking a distance of 2 km). Respondents were asked if they could do each of these activities easily, with difficulty or could not do the activity at all. If a respondent indicated ‘with difficulty’ or ‘not at all’ for any of these activities, she was classified as having an ADL difficulty. All health outcomes are coded such that a positive value indicates an adverse health outcome.

The second set of outcomes examined relate to fertility, contraception and child mortality. Fertility-related outcomes examined included age at first birth and adolescent childbearing (giving birth to a child before age 20). We also examined current use of modern contraceptives and unmet need for contraceptives (defined as when a woman reported not wanting any more children but was not using any contraceptive). Modern contraceptives were defined as female/male sterilization, intrauterine device (IUD), injectable, implant, pill, male/female condom, diaphragm, or foam/jelly. Traditional methods were lactational amenorrhoea method (LAM), periodic abstinence or withdrawal and were also considered for the ‘any contraceptives’ indicator. Finally, child mortality was examined for the first-born child.^7^

Next, we assessed the savings behaviour of the women, as a proxy for their control over household resources. We asked whether women were currently saving any money in cash and created a variable equal to one if a respondent reported to be currently saving money and equal to zero otherwise.

Stress was measured by the Perceived Stress Scale (PSS), consisting of ten questions, six negatively phrased and four positively phrased assessing the degree to which individuals experience their lives as unpredictable, uncontrollable and overloading [30]. The PSS has been validated in countries across the world and is increasingly being used in Sub-Saharan Africa [31–33], though has not been validated in the African setting to date. Responses included Likert-type options ranging from 0 (never) to 4 (very often/always). The scale was constructed by first reverse coding positive items and then adding the total score from each question resulting in a scale ranging from 0 to 50, where a higher score indicates higher stress. Cronbach’s alpha, which tests internal consistency of a set of scale items, for this scale was 0.67.

To measure social support, the survey employed the eight-item modified Medical Outcomes Study Social Support Survey (mMOS-SS) [34]. Items in this scale include questions such as: whether the individual has someone who would help them if they were confined to bed, take them to the doctor if they need it, prepare their meals if they are unable to do it themselves, help with daily chores if they are sick, have a good time with, turn to for suggestions about dealing with a personal problem, someone to understand their problems, and having someone to love and make them feel wanted. Response options on a Likert-type scale ranged from 1 (none of the time) to 5 (all of the time). The score was obtained by averaging the responses to the items and then standardizing them, resulting in a score with a possible range from 0 to 100, where higher scores indicate higher social support levels. Cronbach’s alpha for the eight statements is 0.90.

Finally, women’s agency was operationalized via a locus of control index measured by a Likert scale from 1 (none of the time) to 5 (all of the time). The scale has been loosely based on Rotter’s internal versus external locus of control scale [35], yet we have contextualized it to better match our research population and study objectives. More specifically, we employed the following six statements: Your life is determined by your own actions; You have the power to make important decisions that change the course of your own life; You have the power to make important decisions that change the wellbeing of your children; You have the power to make important decisions that change the wellbeing of your household; You are capable of protecting your own interests within your household; You are capable of protecting your own interests outside of your household (e.g. in the community, in groups in which you participate). Internal consistency among the statements is high, with Cronbach’s alpha of 0.79. The six statements are each dichotomized by coding the indicator as one for response options most/all of the time and zero otherwise. Individual items in the scale have been found to be positively affected by cash and in-kind transfers in Ecuador [36], and an index using similar questions predicted the level of impatience and demand for commitment and saving in rural Ethiopia [37].

### Data analysis

We first summarized background characteristics and outcome indicators. Next, we examined subsequent risks associated with child marriage, by estimating a series of regression models (logistic for dichotomous outcome variables and ordinary least squares (OLS) for continuous outcomes) for each of the outcomes described above. Controls used in this analysis included individual characteristics (age and highest educational attainment), household-level factors (household size; number of members in each of the following age groups: 0 to 5 years, 6 to 12 years, 13 to 17 years, 18 to 24 years, 25 to 34 years, 35 to 44 years, 45 to 54 years, and 55 to 64 years; sex of the head of household; whether the head of household had ever attended school; log of monthly household adult equivalent consumption in Ghana cedi) and a dichotomous variable for each of the districts Karaga, Yendi, Bongo and Garu-Tempane.

We estimated all regressions on two subgroups of women who have ever been married or cohabited. First, we take the typical age group for research on child marriage: 20 to 24 years. Due to relatively small sample size for some of our outcomes in this age group, we also estimate our regressions on a larger subsample of women: 20 to 29 years. Sample sizes for regressions run on different outcomes varies, as information on contraceptive use, empowerment, social support, stress and time preferences were collected among only one women per household (the main survey respondent), while outcomes such as general health and reproductive health are available for all women of reproductive age in the households.

All analyses were run with Stata 14.2 (College Station, TX) and in multivariate analyses, standard errors were adjusted in all analyses to account for clustering at the community-level.

## Results

### Sample characteristics

Table 1 provides descriptive statistics stratified by age group. Educational attainment of the women in the sample is low, with close to 70% attaining no education at all in the 20 to 24 year age group (75% in the 20 to 29 year age group). Most of the women live in the Northern region of Ghana, which is also a reflection of the targeting of the cash transfer programme, and a majority of women are in a monogamous marriage or union. However, polygyny is common, with 25% of woman in the 20 to 24 year age group in a polygamous marriage or union (31% of women aged 20 to 29 years). The child marriage rate in the 20 to 24 year age group is 30%. Approximately 4% of 20 to 24 year olds married before age 15. In the 20 to 29 year age group, approximately one in four women married before age 18 and 3.5% before age 15.^8^

Table 2 depicts the means and standard deviations of the outcomes examined in this study. Approximately one third of the sample (24% of those 20 to 24 years) reports having an illness in the last 2 weeks, and 21% of the sample (18%) self-report being in fair or poor health. Further, 44% (46%) report not having a valid NHIS card. With respect to fertility and contraception, the average age of first birth in our sample was 19 years, and 45% (47%) report having their first birth during adolescence. Rates of modern contraceptive use were 14% (12%). This low rate (compared to 21 to 24% of women aged 20 to 29 years nationally using a modern method [16]) likely reflects both poverty-related contraceptive-access barriers as well as study eligibility criteria (pregnancy and post-pregnancy), when women are less likely to be using contraception. Very few women report currently saving money in cash (6% of the full sample and 4% of the 20 to 24 year olds), reflecting general conditions of poverty in this sample. Further, in the agency (empowerment) scale, the percentage reporting they believe they have the power to make decisions related to the various items range from 11% (household well-being (9%)) to 22% (life course (21%)). Averages on the MOS-SS and Cohen perceived stress scales were 51.99 (53.34) and 21.64 (21), respectively.

**Table 2:** Means and standard deviations of outcome variables, Women aged 20–29 years, Ghana LEAP 1000 Baseline

	Ever-married women 20 to 24 years old			Ever-married women 20 to 29 years old		
	N	Mean	SD	N	Mean	SD
Health outcomes						
Illness in last 2 weeks	594	0.24	0.43	1349	0.30	0.46
No valid NHIS card	594	0.46	0.50	1348	0.44	0.50
Fair/poor self-rated health	516	0.18	0.38	1174	0.21	0.41
Believes health is not better than a year ago	516	0.52	0.50	1174	0.54	0.50
Has difficulty with ADL	516	0.48	0.50	1174	0.50	0.50
Fertility, contraception, and child mortality						
Age of first birth	493	18.93	2.25	1150	19.32	2.71
Adolescence childbearing	594	0.47	0.50	1349	0.45	0.50
First born child died	495	0.08	0.27	1152	0.10	0.30
Use any contraceptive	447	0.12	0.32	1025	0.15	0.36
Use modern contraceptive	447	0.12	0.32	1025	0.14	0.34
Unmet need for contraception	447	0.03	0.17	1025	0.03	0.17
Empowerment, agency, support & stress						
Currently saving money	516	0.04	0.20	1174	0.06	0.24
Believes life determined by own actions	516	0.21	0.41	1174	0.22	0.41
Believes have power to make decisions - life course	516	0.21	0.40	1174	0.22	0.41
Believes have power to make decisions - children’s wellbeing	516	0.14	0.35	1174	0.16	0.37
Believes have power to make decisions - household wellbeing	516	0.09	0.29	1174	0.11	0.32
Believes capable protecting own interests within family	516	0.10	0.30	1174	0.12	0.32
Believes capable protecting own interests outside family	516	0.11	0.31	1174	0.15	0.36
MOS-Social Support score	516	53.34	23.25	1174	51.99	22.74
Cohen perceived stress scale	516	21.00	4.70	1174	21.64	4.94

### Association between child marriage and health outcomes

**Fig. 1:**
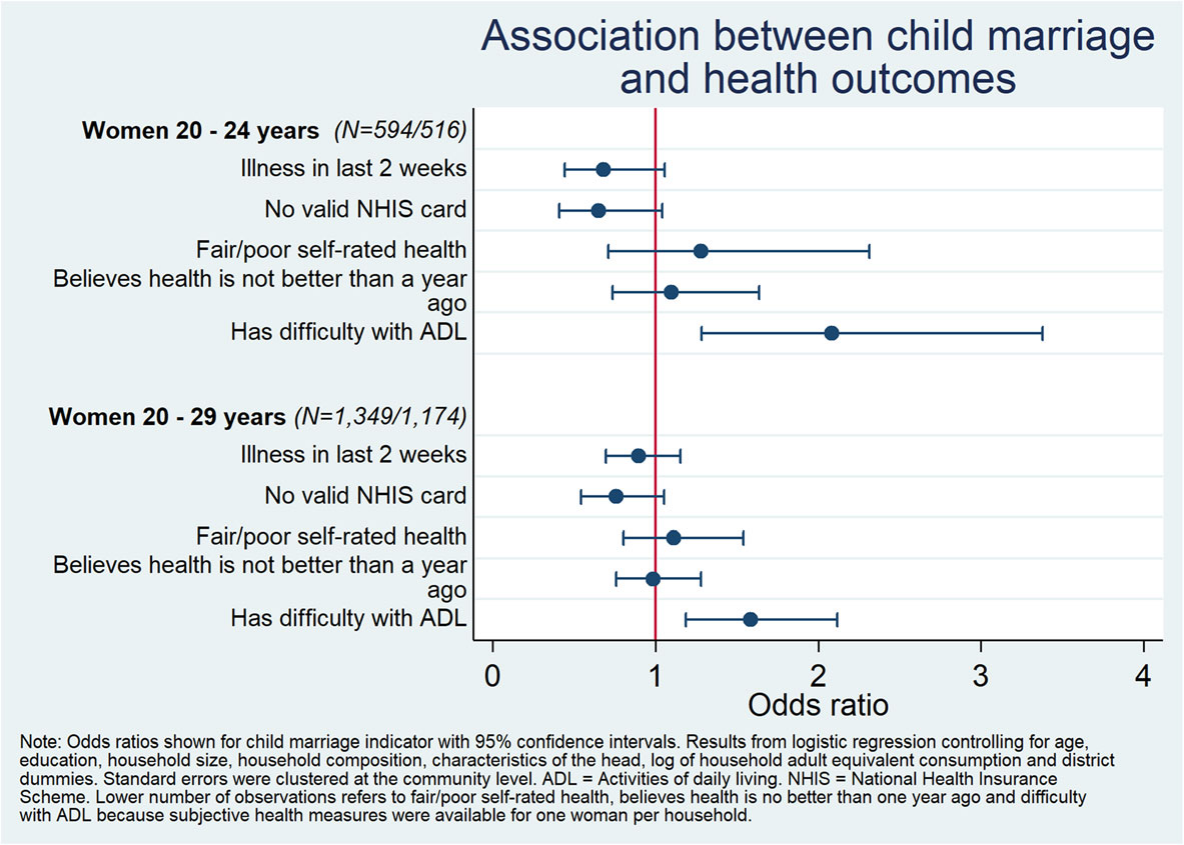
Odds ratios (95% confidence interval) of the association between child marriage and health outcomes, Women 20–29 years, Ghana LEAP 1000 Baseline

Figure 1 presents outcomes related to general health.^9^ Child marriage was associated with increased likelihood of having ADL difficulties (OR = 2.08; CI 1.28–3.38 among women 20 to 24 years and OR = 1.58; CI 1.19–2.12 among women 20 to 29 years). There was no relationship between child marriage and illness, having a valid NHIS card, self-rated health, or self-reported improved health compared to one year prior.

### Association between child marriage and fertility outcomes, contraception and child mortality

**Fig. 2:**
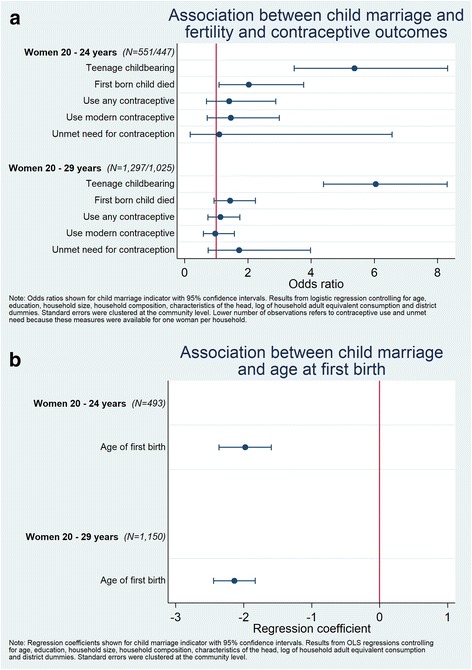
**a** Odds ratios (95% confidence interval) of the association between child marriage and fertility, contraceptive use, and child mortality among women whom ever gave birth, Women 20–29 years, Ghana LEAP 1000 Baseline. **b** Regression coefficients (95% confidence interval) of the association between child marriage and age at first birth, among women whom ever gave birth, Women 20–29 years, Ghana LEAP 1000 Baseline

Results related to fertility are reported in Fig. 2A (dichotomous outcomes) and Fig. 2B (continuous outcomes). Child marriage was associated with earlier childbearing; the mean age at the birth of their first child is approximately two years younger for women who married as children compared to women who married as adults (Regression Coefficient (RC) = − 1.98; CI -2.36 – -1.59 among the 20 to 24 year old sample; RC = − 2.14; CI -2.44 – -1.83 among the 20 to 29 year old sample). This is also reflected in the likelihood of adolescence childbearing, which is much higher among women marrying before age 18 (OR = 4.97; CI 3.15–7.86 among women 20 to 24 years and OR = 5.71; CI 4.06–8.05 among women 20 to 29 years). Further, first-born children of women who married before age 18 had increased odds of child mortality, compared to first-borns of women who married after 18 years (OR = 2.03; CI 1.09–3.77 among women 20 to 24; not statistically significant among women 20 to 29 years). Child marriage was not associated with differences in current contraceptive use, either traditional or modern, or unmet need for contraception.^10^

### Association between child marriage and empowerment, social support and stress

**Fig. 3:**
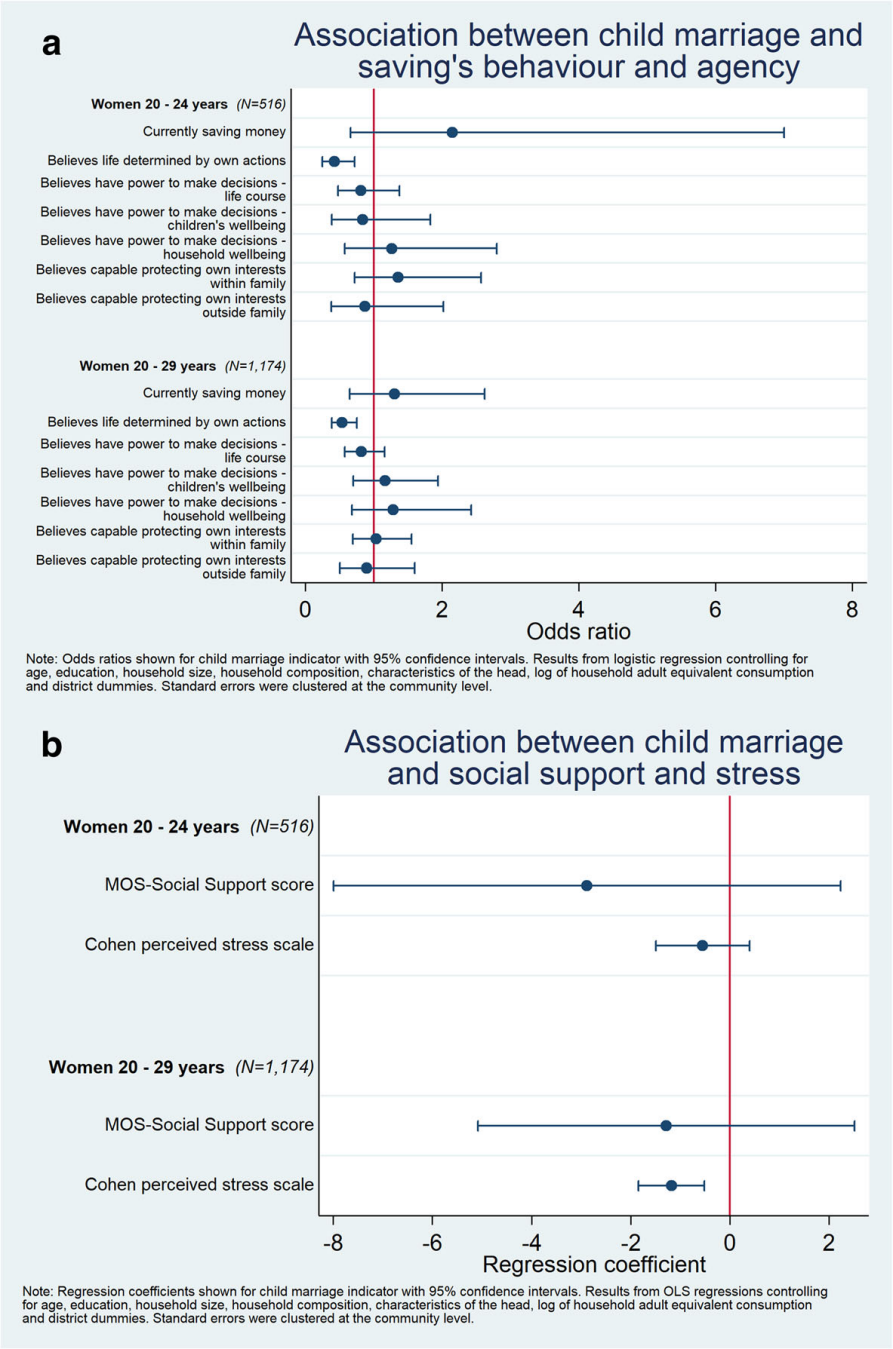
**a** Odds ratios (95% confidence interval) of the association between child marriage and saving’s behaviour and agency, Women 20–29 years, Ghana LEAP 1000 Baseline. **b** Regression coefficients (95% confidence interval) of the association between child marriage and social support and stress, Women 20–29 years, Ghana LEAP 1000 Baseline

In Fig. 3A (dichotomous outcomes) and Fig. 3B (continuous outcomes) we present findings related to savings, support, self-perceived stress and agency. Child marriage was associated with lower levels of reported stress in the 20 to 29 year age group (Coefficient = − 1.18; CI -1.84 – -0.51), but not among those ages 20 to 24 years. In contrast, women married as children had lower odds of believing that their life was determined by their own actions (OR = 0.42; CI 0.25–0.72 among women 20 to 24 years and OR = 0.54; CI 0.39–0.75 among women 20 to 29 years). There was no significant relation between child marriage and saving, social support, or the remaining agency statements in this sample.

## Discussion

In this paper, we examined child marriage rates among an extremely poor population in northern Ghana, a region with among the highest rates of child marriage in the country. We found that child marriage was associated with increased likelihood of difficulties with ADLs, higher likelihood of adolescence pregnancy, a higher likelihood of child mortality among first-born children (only among women 20 to 24 year old) and lower odds of believing that life is determined by one’s own actions. Our unique data allow us to examine outcomes not generally measured in other studies on child marriage, including those related to savings, agency, social support and stress. Nevertheless, we found few associations between child marriage and these outcomes. There was no relationship between child marriage and illness, having a valid NHIS card, self-rated health, self-reported improved health compared to one year prior, current contraceptive use, unmet need for contraception, saving, social support, or the remaining agency statements.

Some of our findings are consistent with evidence from other countries and regions. For example, previous studies have shown significant risks for children born to teenage brides, such as premature birth, low-birth weight and a higher likelihood of dying before their second birthday [6, 10]. The finding that child marriage was associated with adolescent child bearing reflects norms whereby girls and women are expected to start bearing children soon after marriage. We find that child marriage was associated with child mortality among women 20 to 24 years old, and previous studies support the links between child marriage and poor child health [6]. Hence, efforts to improve early childhood nutrition should be linked to programming aimed at reducing child marriage, as well as that addressing nutrition needs of pregnant adolescents or adolescent girls more generally in areas with high rates of early marriage. In this way, initiatives can have beneficial impacts for adolescent girls as well as their future children, helping to break the inter-generational cycle of poverty and poor health. To the best of our knowledge, previous studies have not examined the association between child marriage and ADL difficulties, but a common finding in the literature is that child marriage is associated with poorer general health outcomes [19].

In addition, the finding that child brides were less likely to believe that their life was determined by their own actions was in line with our expectations, given evidence that young wives have typically little bargaining power within the household [19]. Still, our findings on stress may somewhat contradict existing research on mental health, which find that child marriage is associated with poorer mental health, such as a suicidality, lack of self-esteem and depression [7, 38]. However, we are not aware of any studies on child marriage and subsequent stress.

As with any research, there are limitations to this paper. Given the cross-sectional nature of the data (we examine both the self-report of child marriage and outcomes at the same time), we cannot control for the potential endogeneity of the marriage decision (i.e., are there unobserved factors driving both the child marriage decision and the observed outcomes). For example, poorer health outcomes and child marriage may be linked to other common factors such as poverty, social norms and expectations in the “sending” households (that is, households that are making the decision to marry off girls at an early age). Our data only represent “receiving” households, which is not an ideal study design to study impacts of child marriage, but is similar to existing studies in the public health literature examining associations between early marriage and adverse outcomes using cross-sectional data. However, given the homogeneity of the study sample (similar levels of poverty and area of residence) our estimates may better isolate the impacts of early marriage as compared to other studies and more heterogeneous samples, where poverty and related factors may drive both early marriage and adverse outcomes, biasing estimates of these relationships. In addition, the findings reported here are specific to a geographical location and demographic group, namely pregnant women or women with an infant in two Northern regions in Ghana. Thus our findings are not necessarily generalizable to the country as a whole, although many of the results are in line with findings from other studies.

While this paper has demonstrated adverse outcomes associated with child marriage among females, there is limited existing evidence on what works best to prevent child marriage. A scan of programmes in 2007 found that there are relatively few programmes operating around the globe given the scope of the problem, and that there are very few that actually attempt to measure the results in terms of reduction of child marriages [6]. Schooling is linked to reductions in child marriage and therefore interventions that address school drop-out among adolescent girls at the secondary level may also reduce early marriage, as evidence has shown in Zimbabwe [39], and Malawi and Uganda [40]. A recent review paper, reviewing 23 programmes addressing child marriage implemented between 1973 and 2009, found that programmes offering incentives (such as cash transfers or school material) and those attempting to empower girls (by providing information and skills and changing attitudes and practices) can be effective at reducing rates of child marriage [41]. However, the limited evidence available examining the ability of government cash transfer programmes in Africa to reduce early marriage is mixed at best and more research is needed [42, 43]. While the aforementioned research investigates programmes to prevent child marriage, little research is done about what kind of programmes work to help children who are already married. Since this study has shown that early childbirth is a major issue among child brides, programmes that increase access to contraception and family planning services should be promising. In addition, livelihood support programmes can help young brides to generate their own income and increase their agency [44].

In Ghana, several efforts are currently underway to combat child marriage. In 2014, the Ministry of Gender, Children and Social Protection set up an Ending Child Marriage Unit, with the mandate to promote and coordinate national efforts to end child marriage in Ghana. More recently, a national campaign was launched to raise awareness about the issue and the government is working on a 10-year national strategic framework with the primary goal to end child marriages by 2030 [45]. In addition, Ghana is one of the 12 countries participating in the new UNICEF and UNFPA multi-country initiative to accelerate action to end child marriage [46]. While such initiatives and advocacy are an important first step, more research is still needed in Ghana on effective prevention strategies.

## Conclusion

In this study among poor, rural women in Northern Ghana, child marriage was associated with increased difficulty with ADLs, early childbearing, child mortality, lower agency and reduced stress levels. There was no relationship between child marriage and illness, having a valid NHIS card, self-rated health, self-reported improved health compared to 1 year prior, current contraceptive use, unmet need for contraception, saving or social support. These findings support the need for more initiatives aimed at reducing child marriage; however, effective strategies remain elusive. Future research should include studies on the effectiveness of initiatives aimed at mitigating adverse impacts among women and children affected by child marriage as well as efforts around primary prevention.

## Abbreviations

ADL: Activities of Daily Living
CRC: Convention on the Rights of the Child
DHS: Demographic and Health Surveys
HIV/AIDS: Human immunodeficiency virus infection and acquired immune deficiency syndrome
ISSER: Institute of Statistical, Social and Economic Research
IUD: Intrauterine Device
LAM: Lactational Amenorrhoea Method
LEAP: Livelihood Empowerment Against Poverty
MICS: Multiple Indicator Cluster Surveys
mMOS-SS: Modified Medical Outcomes Study Social Support Survey
NHIS: National Health Insurance Scheme
OLS: Ordinary Least Squares
OR: Odds Ratio
PSS: Perceived Stress Scale
UNFPA: United Nations Population Fund
UNICEF: United Nations Children’s Fund

## Footnotes

1 Maternal disorders in the Global Burden of Disease report include: maternal haemorrhage; maternal sepsis and other maternal infections; maternal hypertensive disorders; maternal obstructed labour and uterine rupture; maternal abortion, miscarriage and ectopic pregnancy; indirect maternal deaths; late maternal deaths; maternal deaths aggravated by HIV/AIDS; and, other maternal disorders.

2 All figures in this paragraph are based on authors’ calculations from the Ghana Multiple Indicator Cluster Surveys and Ghana Demographic and Health Surveys.

3 We define adolescents as those between 10 and 19 years of age [47].

4 The total sample size for the evaluation was established at 2500 households. Hence, the response rate to the survey was nearly universal.

5 The LEAP programme uses a proxy means test (PMT) with a sharp threshold to determine if households meet the poverty criterion. The PMT takes into account household demographic characteristics, assets and housing conditions. The evaluation is a ‘regression discontinuity design’. As such, about 50% of the sample consists of households just below the threshold with the other 50% of households scoring above the poverty threshold. For more information about the study design and the sampling, see [27].

6 We also considered disability, but the share of women who reported a disability in our sample is very low (1%) and the estimates are therefore unreliable.

7 We only consider first-born children here because child brides start bearing children earlier, and hence have more children at the time of the survey and thus higher chance that at least one of their children has died.

8 Since our sample is not a random sample of the population, this does not necessarily mean that the rate of child marriage has been increasing.

9 We display our findings graphically, for ease of reading. Full regression output is available from the authors upon request.

10 The confidence interval for unmet need for contraception is large, due to the low number of women with an unmet need (approximately 3%). This low value is not uncommon, as the DHS value of a similar indicator is 2.0% for women 20 to 24 years old and 5.0% for women 25 to 29 years old [16].
